# Impact of rare and common genetic variation in the interleukin-1 pathway on human cytokine responses

**DOI:** 10.1186/s13073-021-00907-w

**Published:** 2021-05-25

**Authors:** Rosanne C. van Deuren, Peer Arts, Giulio Cavalli, Martin Jaeger, Marloes Steehouwer, Maartje van de Vorst, Christian Gilissen, Leo A. B. Joosten, Charles A. Dinarello, Musa M. Mhlanga, Vinod Kumar, Mihai G. Netea, Frank L. van de Veerdonk, Alexander Hoischen

**Affiliations:** 1 Department of Internal Medicine, Radboud Expertise Center for Immunodeficiency and Autoinflammation, and Radboud Center for Infectious Disease (RCI), Radboud University Medical Center, Nijmegen, the Netherlands; 2 Department of Human Genetics, Radboud University Medical Center, Nijmegen, the Netherlands; 3 Radboud Institute of Molecular Life Sciences (RIMLS), Radboud University Medical Center, Nijmegen, the Netherlands; 4 Department of Genetics and Molecular Pathology, Centre for Cancer Biology, SA Pathology and the University of South Australia, Adelaide, South Australia Australia; 5 Unit of Immunology, Rheumatology, Allergy and Rare Diseases, IRCCS San Raffaele Hospital and Vita-Salute San Raffaele University, Milan, Italy; 6 Department of Medicine, University of Colorado, Aurora, CO USA; 7 Department of Medical Genetics, Iuliu Hatieganu University of Medicine and Pharmacy, Cluj-Napoca, Romania; 8 Epigenomics & Single Cell Biophysics Group, Department of Cell Biology, Radboud University, 6525 GA Nijmegen, The Netherlands; 9 Department of Genetics, University of Groningen, University Medical Center Groningen, Groningen, The Netherlands; 10 Nitte (Deemed to be University), Nitte University Centre for Science Education and Research (NUCSER), Medical Sciences Complex, Deralakatte, Mangalore, 575018 India; 11 Department for Genomics & Immunoregulation, Life and Medical Sciences Institute (LIMES), University of Bonn, Bonn, Germany

**Keywords:** Rare variants, SKAT, Common variants, Region-based analysis, Interleukin-1 pathway, Immunological mechanisms, Systems biology

## Abstract

**Background:**

The interleukin (IL)-1 pathway is primarily associated with innate immunological defense and plays a major role in the induction and regulation of inflammation. Both common and rare genetic variation in this pathway underlies various inflammation-mediated diseases, but the role of rare variants relative to common variants in immune response variability in healthy individuals remains unclear.

**Methods:**

We performed molecular inversion probe sequencing on 48 IL-1 pathway-related genes in 463 healthy individuals from the Human Functional Genomics Project. We functionally grouped common and rare variants, over gene, subpathway, and inflammatory levels and performed the Sequence Kernel Association Test to test for association with in vitro stimulation-induced cytokine responses; specifically, IL-1β and IL-6 cytokine measurements upon stimulations that represent an array of microbial infections: lipopolysaccharide (LPS), phytohaemagglutinin (PHA), *Candida albicans* (*C. albicans*), and *Staphylococcus aureus* (*S. aureus*).

**Results:**

We identified a burden of *NCF4* rare variants with PHA-induced IL-6 cytokine and showed that the respective carriers are in the 1% lowest IL-6 producers. Collapsing rare variants in IL-1 subpathway genes produces a bidirectional association with LPS-induced IL-1β cytokine levels, which is reflected by a significant Spearman correlation. On the inflammatory level, we identified a burden of rare variants in genes encoding for proteins with an anti-inflammatory function with *S. aureus*-induced IL-6 cytokine. In contrast to these rare variant findings which were based on different types of stimuli, common variant associations were exclusively identified with *C. albicans*-induced cytokine over various levels of grouping, from the gene, to subpathway, to inflammatory level.

**Conclusions:**

In conclusion, this study shows that functionally grouping common and rare genetic variants enables the elucidation IL-1-mediated biological mechanisms, specifically, for IL-1β and IL-6 cytokine responses induced by various stimuli. The framework used in this study may allow for the analysis of rare and common genetic variants in a wider variety of (non-immune) complex phenotypes and therefore has the potential to contribute to better understanding of unresolved, complex traits and diseases.

**Supplementary Information:**

The online version contains supplementary material available at 10.1186/s13073-021-00907-w.

## Background

The innate immune system is our first line of defense against invading pathogens and is shaped by a well-maintained balance in stimulatory and inhibitory mechanisms [1]. The interleukin (IL)-1 family of cytokines and receptors is primarily associated with innate immunity and plays a major role in the induction and regulation of host defense and inflammation [2]. The IL-1 family comprises pro-inflammatory cytokines (*e.g.*, IL-1α/β, IL-36α/β/γ), anti-inflammatory cytokines (*e.g.*, IL-37, IL-38), activating receptors (*e.g.*, IL1-R1, IL-36R), decoy receptors (*e.g.,* IL-1R2, IL-18BP), and additional regulators, kinases, and phosphatases that together are responsible for the IL-1-mediated response [3]. Next to core IL-1 family effectors, members of the inflammasome and autophagy pathway are important contributors to the regulation of IL-1-induced inflammation. For instance, activation of the inflammasome allows for cleavage and activation of CASP-1, with subsequent activation and release of pro-inflammatory cytokines IL-1β and IL-18. Conversely, autophagy is able to directly inhibit the inflammatory response by removing inflammasome components and damaged mitochondria [4].

Defects in IL-1 pathway signaling and its specific members have been linked to various inflammation-mediated diseases [2, 5, 6]. Generally, the clinical presentation of dysregulated activity of the IL-1 pathway is clearly explained by the causal genetic defect. For example, patients with CAPS (cryopyrin associated periodic syndromes) present with excessive innate inflammation exacerbations that appear to be caused by an activating mutation in *NLRP3* resulting in an overproduction of IL-1β [5]. In another example, deleterious mutations in *IL1RN* were underlying excessive IL-1α/β activity in patients with DIRA (deficiency of IL-1 receptor antagonist [7]. Contrastingly for some diseases, like adult-onset Still’s Disease (AoSD), Behcet’s disease, and Schnitzler disease, only subsets of patients have presented with mutations in related genes [7]. Taken together, this underlines the observation that no causal genetic defect has been identified that explains all patients, despite clinical similarities with other inflammation-mediated diseases, like CAPS.

While the IL-1 pathway has been associated with disease, not much is known about genetic factors that can explain immune variability in healthy individuals. In general, immune responses are highly variable between individuals. Determining the genetic factors that underlie these variations in immunological response could be instrumental in the generation of targeted hypotheses for genetic studies in inflammatory diseases that are outside the spectrum of healthy immune variability. For this reason, in the past few decades, various studies have assessed the separate and shared contribution of host and environmental factors to an immunological response after a specific stimulus [8–12]. However, a considerable percentage of “healthy immune response variation” between individuals remains unexplained, with one important shortcoming being that most studies to date have focused on common genetic variants. Unfortunately, this has left the impact of rare or private variants on healthy immune variability poorly understood. With recent advancements in sequencing technologies, the ability to study the role of rare variants has remarkably improved, and its value has been proven in several studies. Increasing evidence shows that variability in phenotypic presentation can be explained by an interplay between variants of variable frequencies [13, 14], or aggregation of genetic variants in genes underlying dysregulated biological mechanisms, or even over genes that are more distantly involved [15]. The relatively small-to-moderate effects of common variants can be significantly modified by the presence or absence of (multiple) rare variants [16]. We therefore hypothesize that studies on the genetic basis of inflammatory diseases or healthy immune variability might also benefit from these concepts.

**Fig. 1:**
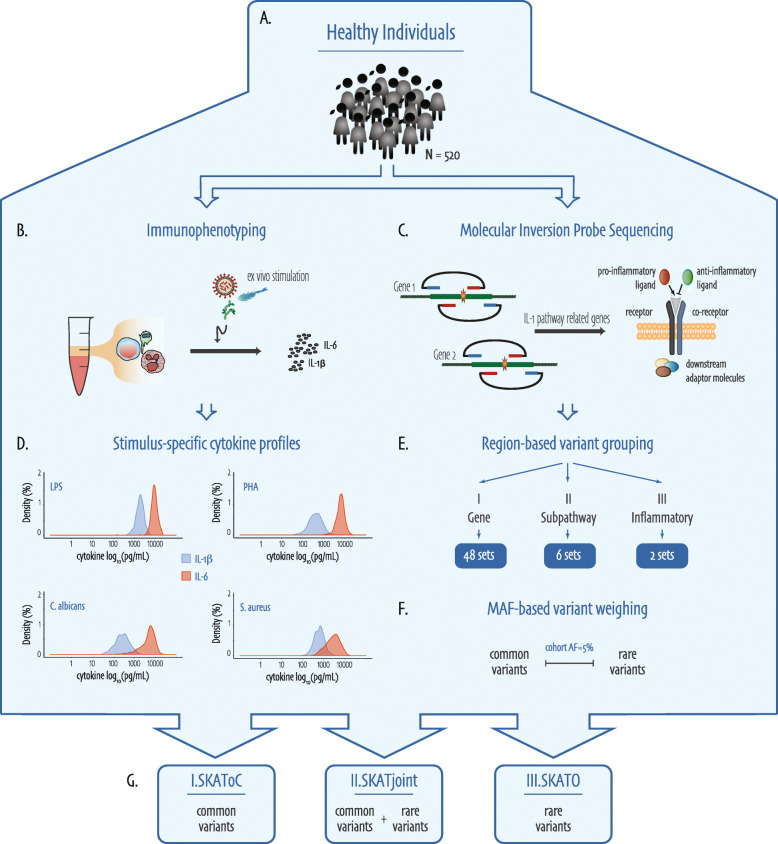
Flowchart of the study. Figure orientation from top to bottom. **a** Blood was extracted from 520 healthy individuals on which (**b**) extensive immunophenotyping was performed and simultaneously (**c**) molecular inversion probe sequencing data was produced from the coding regions of 48 Interleukin-1 pathway-related genes. **d** The resulting cytokine production after stimulation was measured and log-transformed prior to analysis. **e** The identified variants were grouped over three different levels into sets based on gene-encoded protein function: I. Gene level, with 48 genes; II. Subpathway level, grouping genes into 6 subpathways that represent an immunological cascade in the IL-1-mediated inflammatory response; and III. Inflammatory level, with two groups that distinguish between pro- and anti-inflammatory roles of the respective gene-encoded proteins. **f** Variants within each set were appropriately weighed based on minor allele frequency (MAF), and common and rare variants were classified based on cohort allele frequency (AF) threshold of 5%. **g** Finally, variant analysis was performed by the Sequence Kernel Association Test (SKAT) on only common variants (I.SKAToC); common and rare variants combined (II.SKATjoint); and only rare variants using the best combination of the SKAT and burden test (III.SKATO)

In this study, we aimed to identify and characterize rare and common genetic variants in 48 genes related to the IL-1 pathway-mediated immune response and determine their impact on the inter-individual variability of cytokine responses in healthy individuals. A complete overview of the study workflow can be found in Fig. 1.

## Methods

### Study cohort

#### Cohort characteristics

The study was conducted using healthy individuals from the Human Functional Genomics Project (HFGP; 500FG cohort) [17]. The entire 500FG cohort consists of 534 healthy individuals from the Netherlands (296 females and 237 males) with an age range 18–75, from which we were able to obtain DNA from 520 individuals for sequencing. For more details on cohort characteristics, see previous publications on the 500FG cohort [8, 9, 11].

#### Immunophenotyping

Here, we made use of the publicly available extensive immunophenotyping data that was generated as part of the Human Functional Genomics Project [18]. Specifically, interleukin-1β (IL-1β) and interleukin-6 (IL-6) production by whole blood (consisting mainly of polymorphonuclear cells (PMNs)) from 471 individuals, stimulated with either lipopolysaccharide (LPS, 100 ng/mL), phytohaemagglutinin (PHA, 10 μg/mL), heat-killed *Candida albicans* (*C. albicans* 10^6^ CFU/mL), or *Staphylococcus aureus* (*S. aureus* 1 × 10^6^/mL). A detailed description of these experiments can be found elsewhere [9]. In brief, blood was drawn from participants and 100 μL of heparin blood was stimulated with 400 μL of stimulus, subsequently incubated for 48 h at 37 °C and 5% CO_2_ and supernatants were collected and stored in − 20 °C until cytokine measurements were performed by ELISA. Cytokine production by whole blood (consisting of a mix of immune cell subtypes) is most comparable to the in vivo situation, as the cross-regulation between different cell types is very important in determination of the final immune response. The investigated stimuli were chosen as representatives for an array of microbial infections, specifically, LPS is expressed on the bacterial cell wall of Gram-negative bacteria, PHA is synthesized by *Bacillus Rhodococcus* and *Pseudomonas species*, and *C. albicans* and *S. aureus* are major invading pathogens representative of fungi and Gram-positive bacteria, respectively.

### Sequencing

#### MIP panel design

We sequenced all coding exons of 48 genes of the IL-1 pathway in 520 healthy individuals by Molecular Inversion Probe (MIP) sequencing, a targeted resequencing technology that allows for the identification of both common and rare genetic variation in regions of interest. A detailed description of MIP probe design and sequencing methods can be found elsewhere [19–21]. In short, 1285 MIP probes were designed to cover all coding exons of 48 genes related to the IL-1 pathway and sequencing was performed using the Illumina NextSeq500 system. These 48 IL-1 pathway-related genes were chosen for their effector (*e.g.*, *IL1A/B*, *IL36A/B/G*, *IL38*), regulatory (*e.g.*, *IL1RN*, *IL18BP*), and modulatory (*e.g.*, *NLRP3*, *NCF4*, *ATG16L1*) roles in the innate immune response. They can be further functionally subclassified into six subpathways that represent a specific modulatory mechanism or immunological cascade in the IL-1-mediated inflammatory response: IL-1 subpathway, IL-18 subpathway, IL-30s subpathway, inflammasome, reactive oxygen species (ROS) production, and autophagy. In addition, distinguishing between pro- and anti-inflammatory roles of the respective gene-encoded proteins resulted in a third sub-classification of two inflammatory groups. A full explanation on the sub-classifications can be found in Additional file 1: Table S1.

#### Data processing

A carefully developed filtering pipeline, validated by Sanger sequencing, was applied to ensure high sensitivity and specificity in our final variant set. First, the reads were aligned using BWA-MEM [22] and subsequently filtered on Mapping Quality ≥ 60, no soft-clipping, properly paired and less than five mismatches from the reference per read, with the exception of multi-basepair insertions and deletions. Variants were then called using the Genome Analysis Toolkit (GATK) unified genotyper [23], which uses a Bayesian genotype likelihood model to estimate the most likely genotypes. Rare variants (here defined as absent in dbSnp build 150 common [24], or defined as rare by our custom annotator as explained below), were further filtered on the QUAL parameter ≥ 1000 in the vcf. Additionally, the percentage of alternative alleles for each variant position was determined using samtools mpileup [25], with maximum read depth 10,000, no BAQ, a minimal mapping quality of 20, and a minimal base quality of 30. Homozygous rare variants required an alternative allele percentage of ≥ 90%, heterozygous an alternative allele percentage of ≥ 25% and < 90%, and an alternative allele percentage of < 25% was considered false positive. The final variant set was annotated using our custom annotator, which makes use of several annotation sources, among others the Variant Effect Predictor from Ensembl [26], Combined Annotation Dependent Depletion score [27], SpliceAI [28], and several population-based variant databases (*e.g.*, dbSnp, ExAc and gnomAD [29]) and an “inHouse” database consisting of > 25,000 clinical exomes run at the diagnostic division of the Department of Human Genetics of the Radboud University Medical Center (Radboudumc). We used within-cohort allele frequencies (AFs) to separate rare and common variants, based on a common variant cut-off of ≥ 5%. Samples with an average coverage depth of all MIPs ≥ 100× were included for analysis.

### Variant analysis

#### Continuous trait analysis

A rare variant burden analysis (RVBA) was performed on the log-transformed cytokine levels by using the Sequence Kernel Association Test (SKAT) [14, 30] in R version 3.5.2. The SKAT is a kernel-based test method that aggregates weighted individual variant-score test statistics while allowing variant-variant interactions and is extremely powerful when a genetic region has both protective and deleterious variants or many non-causal variants [14, 30, 31]. The SKAT was performed over three levels of grouping: (I) *gene level*, where all variants in a gene region are combined into a set (Fig. 1e.I), (II) *subpathway level*, where all variants in genes that belong to the corresponding subpathway are combined into a set (Fig. 1e.II), and (III) *inflammatory level*, where based on gene-encoded protein function genes are classified with either a pro- or anti-inflammatory phenotype and all variants from genes in either groups are combined into a set (Fig. 1e.III). All variant sets were pruned for linkage disequilibrium (LD) based on within-cohort metrics and the commonly used *R*^2^ cut-off of > 0.8, using the snpStats package in R [32]. For each region, we used the SKAT_CommonRare function with default weights to determine the effect of only common (I.SKAToC) and combined common and rare variants (II.SKATjoint), and the SKAT-O algorithm with default weights (III.SKATO) to determine the effect of only rare variants, where common and rare variant classification was based on a cohort MAF of 5% (Fig. 1f,g). The SKAT-O algorithm uses a linear combination of the SKAT and Burden Test, making it slightly more powerful than the “normal” SKAT when rare variants in a set are truly causal or influencing the phenotype in the same direction [31]. SKATO accompanying rho-values can be used to assess the contribution of SKAT versus Burden Test for significant sets, reflecting the proportion of bi- and unidirectionality of an association. In the case of rare and joint tests, output based on > 1 variant was considered, and in the case of joint tests, the presence of both rare and common variants in the set was an additional requirement. *P* values were Bonferroni-adjusted for each previously defined test separately, based on the number of groups tested within one level of grouping for each cytokine. For data wrangling and visualizations, we used a variety of R packages, *e.g.*, dplyr, reshape2, ggplot2, scales, ggpubr, ggrepel, hash, ggpmisc, and devtools, all of which are freely available online [33, 34].

#### Validation

We applied stringent Bonferroni adjustment within each analysis group; due to this stringency, we did not apply additional corrections over the different grouping levels (*i.e.*, gene level, subpathway level, inflammatory-phenotype level), nor for the different variant frequency tests (*i.e.*, SKAToC, SKATjoint, SKATO). Instead, we performed 10,000 permutations on all of our significant results to provide additional substantiation for our findings, using the resampling option built into the SKAT package with method “bootstrap.”

In addition, to rule out possible detection bias concerning rare variants due to gene size, gene-specific coverage or sequencing context, we retrospectively assessed the association between synonymous variants and cytokine production upon stimulation, and similarly applied Bonferroni adjustment based on the number of groups tested within one level of grouping for each cytokine separately.

Finally, we performed a binary association analysis on outlier individuals, here defined as extreme cytokine producers. As research has shown that individuals with outlier expression patterns are likely to be enriched in rare variants [35, 36], we hypothesized that outlier individuals with extreme cytokine levels could similarly be enriched in rare variants in specific genes, thereby favoring the identification of stimulus-specific mechanisms. For this purpose, we defined for each cytokine stimulus, the 1% extreme cytokine producers (rounded up, so generally ± 5 individuals), resulting in two groups that were subjected to binary trait association. Specifically, for each cytokine-stimulus combination, the SKATO was applied twice: (1) 1% highest cytokine producers versus all other individuals, and (2) 1% lowest cytokine producers versus all other individuals. In two cases, *C. albicans*-induced IL-1β production low-producers and LPS-induced IL-6 production high-producers, no distinctive categories could be created due to equal cytokine measurements at the 1% cut-off, and as such, the groups were extended to 7 and 9 respectively. Bonferroni adjustment based on the number of groups tested within one level of grouping for each cytokine separately was applied.

#### Follow-up of significantly associated sets

In order to give meaning to our detected associations, we extracted the residual (corrected for covariates age and sex) cytokine production from the SKAT null-model and correlated those to the genotype categories, where applicable. For set-based unidirectional rare variant associations, we correlated the residual cytokine production to rare variant carrier status, whereas for bidirectional associations, we calculated a set-based allelic score based on the rare variants from the respective set. An allelic score is a way to collapse multidimensional genetic data associated with a risk factor into a single variable [37]. We slightly adapted the allelic score calculation to our SKAT-based test results, into a weighted (using the Beta.Weights function from SKAT package), directional (increasing or decreasing cytokine production over the genotype categories) allelic score. Specifically, we inferred the direction of each variant in a set, and combined this with the computed variant weight, by inverting the weight only for variants with decreasing cytokine production over the genotype categories. Genotypes were converted to dosages and multiplied by their directional weight, which was summed up to an allelic score per set of variants. The weighted, directional allelic score was plotted in correlation with the residual cytokine production, including a linear regression line using geom_smooth with method = “lm” with standard error of 0.95, and non-parametric Spearman R with accompanying *P* value were extracted. Of note, as we are not able to incorporate variant-variant interactions into our customized allelic scores, the resulting score will most likely be slightly weaker as compared to the SKAT output. In the case of common variant associations for sets ≤ 2 variants, we correlated cytokine production to genotype categories; homozygous reference, heterozygous, and homozygous variant genotype. Differences in residual cytokine production over the genotype categories were assessed by means of Wilcoxon rank sum test with Bonferroni adjustment for the three tests performed, a *P* value < 0.05 was considered significant. For significant common variant associations based on sets > 2 variants, the same customized allelic score was computed based on all common variants in the respective set.

Additionally, considering accumulating evidence for a role of non-coding genetic variation in health and disease [38, 39], we followed up on common coding variant associations of our study by using the publicly available genotype data from the 500FG cohort, generated with the commercially available SNP chip Illumina HumanOmniExpressExome-8v1.0 (for further details, we refer to previously published work [9, 40]). We extracted all common variants (based on cohort AF ≥ 5%) within NCBI RefSeq “Whole Gene” gene regions and extended the start position by 50 kB upstream [41] for the following sets: *IL36A*, *IL38*, IL-30s subpathway, pro-inflammatory, and anti-inflammatory. Variant sets were pruned for LD as described before, and subjected to the same SKAT with default weights, to test for association with continuous IL-1β (*n* = 428) and IL-6 (*n* = 425) cytokine production. We applied Bonferroni adjustment for the number of sets tested in this follow-up. Significant non-coding common variant sets were collapsed into a set-based weighted, directional allelic score (calculated as described before) and correlated to residual cytokine levels. In addition, to evaluate the individual contribution of non-coding common variants in a set, we computed per SNP linear models using *C. albicans*-induced residual cytokine production as the criterion variable and the SNP in question as predictor variable. The individual SNP effect estimates (or Beta-estimates) were organized by direction and annotated based on their significance. The predictive capacity of the linear models, as reflected by the model *P* value, combined with the magnitude of the Beta-estimate, were used as measures for impact of a specific SNP on cytokine production and as such prioritized rs80339050 for more in-depth follow-up. To gain insights into effects of non-coding SNPs, we used a bioinformatic pipeline to map and analyze transcription factor binding sites within genomic compartments (TADs) using UCSC Genome Browser, and checked public expression Quantitative Trait Loci (eQTL) databases for other immune-mediated correlations of the same variants (https://immunpop.com) [42].

## Results

### Study cohort

In this study, we focused on healthy individuals from the Human Functional Genomics Project (HFGP; 500FG cohort) [17], by making use of the publicly available demographic data and stimuli-specific in vitro cytokine measurements [18]. The sex distribution over 463 included individuals for analysis shows a minor overrepresentation of females as compared to males (male *n* = 201, female *n* = 262), whereas the mean and median age distribution for these groups separately is comparable (Additional file 2: Fig. S1A).

In vitro IL-1β and IL-6 cytokine production in whole blood in response to stimulation with either 100 ng/mL lipopolysaccharide (LPS), 10 μg/mL phytohemagglutinin (PHA), heat-killed *Candida albicans* 10^6 ^CFU/mL (*C. albicans*), and 1 × 10^6^/mL *Staphylococcus aureus* (*S. aureus*) were likewise evenly distributed between females and males (Additional file 2: Fig. S1B) and were log-transformed prior to analysis. Based on the abovementioned distributions, in combination with the fact that previous research has shown that age and sex can influence cytokine responses [8–11], both variables were included as covariates in our analyses.

### Sequencing

Molecular Inversion Probe (MIP) sequencing of all coding exons of the 48 genes in our IL-1 pathway MIP panel generated sequencing data from 520 healthy individuals (for all MIP probes, see Additional file 1: Table S2). Overlapping sequencing data with the available immunophenotyping data, we managed to obtain a complete dataset from 463 individuals for analysis. The average coverage depth for these 463 individuals over all MIPs was 830× (Additional file 2: Fig. S2). Five genes in our panel (*SIGIRR*, *PYCARD*, *CYBA*, *RAC2*, and *MAP1LC3A*) were unfavorably covered for more than half of the samples (< 100× average coverage for the entire coding part of the gene), and one gene (*NCF1*) lost all coverage in our extensive quality filtering due to homology regions and was therefore excluded from all analyses (Additional file 2: Fig. S2). Based on gene-encoding protein function and the immunological cascade in which they are activated, we classified these 48 genes prior to analysis into (1) six subpathway groups: IL-1 subpathway, IL-18 subpathway, IL-30s subpathway, inflammasome subpathway, ROS-production subpathway and autophagy subpathway; and (2) two inflammatory groups: pro-inflammatory and anti-inflammatory (Additional file 1: Table S1).

Overall, we identified 201 non-synonymous variants in the coding regions, out of which 35 were common and 166 were rare (based on cohort allele frequencies (AFs) using a threshold of ≥ 5% for common variants). Our common variants were pruned for linkage disequilibrium (LD) prior to analysis, resulting in 26 non-synonymous common variants and 166 rare variants, of which 18 were novel (*i.e.*, absent from public databases). For a complete variant list, see Additional file 1: Table S3.

### Variant analysis on gene, pathway, and inflammation levels

The role of rare and common variants on stimuli-specific cytokine responses was assessed by a rare variant burden analysis using SKAT. We performed the SKAT using three different grouping strategies (Fig. 1e and Additional file 1: Table S1): (I) gene level, where all variants per gene are combined into a set; (II) subpathway level, where all variants in genes that belong to the corresponding subpathway are combined into a set; and (III) inflammatory level, where based on gene-encoded protein function genes are classified with either a pro- or anti-inflammatory phenotype and all variants from genes in either groups are combined into a set. Each level was assessed for the role of rare and common genetic variants in a set on cytokine production. Output from all SKATs performed in this study can be found in Additional file 1: Table S4, S5, S6.

We applied Bonferroni correction to all SKAT *P* values, and additionally performed a threefold validation to further substantiate our findings. All of our described associations persisted when running them with 10,000 permutations (Additional file 1: Table S7). Moreover, for none of our significant non-synonymous variant SKATO associations, we observed significant differences upon testing for association with synonymous variants in the same group level (for a complete synonymous variant list see Additional file 1: Table S8, for SKAT output, see Additional file 1: Table S9). And finally, we were able to replicate our unidirectional gene level rare variant associations in extreme cytokine producers (Additional file 1: Table S10).

**Fig. 2:**
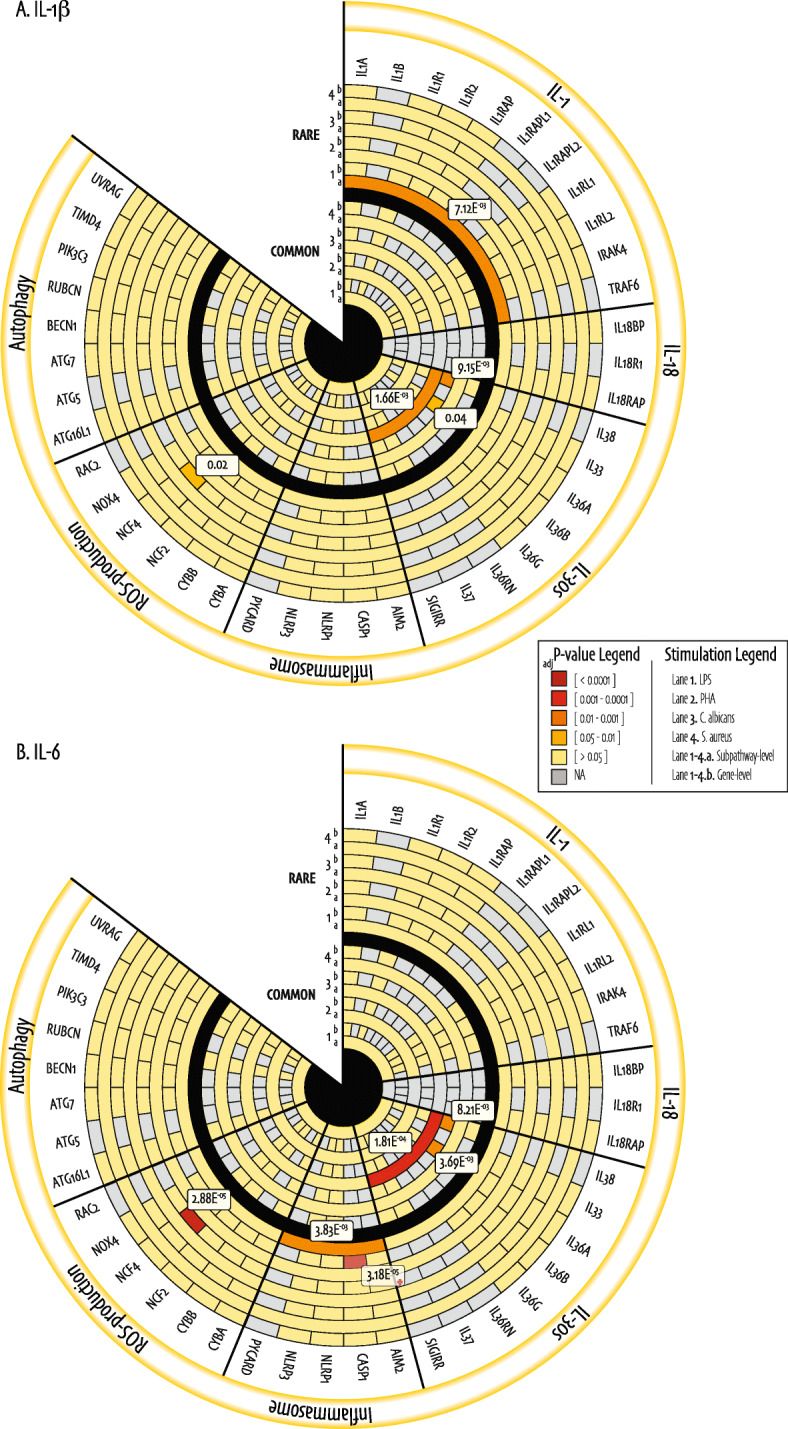
Association landscapes of SKAT (Bonferroni-adjusted) _adj_*P* values. The circular heatmaps consist of two rings separated by a black lane, where the inner ring shows the SKAT _adj_*P* values with only common (AF ≥ 5%) variants (SKAToC), and the outer ring the SKATO _adj_*P* values with only rare (AF < 5%) variants on the gene and subpathway levels with log_10_-transformed IL-1β (**a**) and IL-6 (**b**) cytokine production respectively. Each ring consists of 8 lanes that represent different stimuli; (1) LPS, (2) PHA, (3) *C. albicans*, (4) *S. aureus*, (a) showing the subpathway-level result and (b) the gene-level result. The gene names at the surface of the outer ring of the heatmap are grouped based on corresponding subpathway as annotated on the yellow border, and genes without identified genetic variants are not shown. Genes or subpathways without identified genetic variants contributing to a particular association (*i.e.*, for a gene with a single rare variant the gene-level output is not considered, but it does contribute to the subpathway-level association) have been assigned the value NA and are shown in light gray. Significance of *P* values is highlighted in color, and only significant *P* values are labelled. Annotation: * = the *CASP1* set-based association was the only one not confirmed by Spearman correlation. Abbreviations: SKAT = Sequence Kernel Association Test; LPS = Lipopolysaccharide; PHA = Phytohaemagglutinin; *C. albicans* = *Candida albicans*; *S. aureus* = *Staphylococcus aureus*

**Fig. 3:**
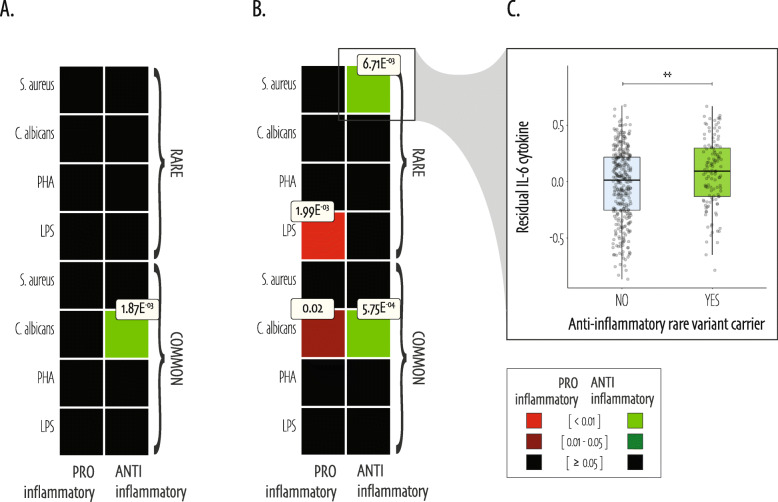
Inflammatory-level cytokine association heatmap SKAT (Bonferroni-adjusted) _adj_*P* values. A heatmap representation of SKAT _adj_*P* values testing for association between variants in pro- or anti-inflammatory sets and IL-1β (**a**) and IL-6 (**b**) cytokine production in response to four different stimuli; LPS, PHA, *C. albicans*, and *S. aureus*. Common and rare variants were tested separately (based on a cohort allele frequency threshold of 5%), by means of the SKAT for common variants and the SKATO for rare variants, in two inflammatory-level groups that distinguish between pro- and anti-inflammatory roles of the respective gene-encoded proteins. Significance of _adj_*P* values is highlighted in color, and only significant _adj_*P* values are labelled. **c** Zooms in on the details of the significant association between *S. aureus*-induced IL-6 cytokine production and anti-inflammatory rare variants. The residual (corrected for age and sex) *S. aureus*-induced IL-6 cytokine production shown on the *y*-axis, is higher in anti-inflammatory rare variant carriers as compared to non-carriers (NO = individuals without rare variant in the anti-inflammatory group; YES = individuals carrying a rare variant in the anti-inflammatory group as shown on the *x*-axis). The Wilcoxon rank-sum *P* value reveals a significant difference between the two categories (*P* value = 0.003). Annotation: ** = < 0.01. Abbreviations: SKAT = Sequence Kernel Association Test; LPS = Lipopolysaccharide; PHA = Phytohaemagglutinin; *C. albicans* = *Candida albicans*; *S. aureus* = *Staphylococcus aureus*

We created holistic heatmap overviews termed “association landscapes,” to summarize rare and common variant associations, both on the gene and subpathway level, in an organized fashion. Figure 2 shows these landscapes of gene and subpathway level associations for IL-1β (Fig. 2a) and IL-6 (Fig. 2b) cytokine production by whole blood, for genes that harbor common or rare variants contributing to the association only. Figure 3 shows the inflammatory-level associations for IL-1β (Fig. 3a) and IL-6 (Fig. 3b) production by whole blood in classic rectangular heatmaps. Of note, we did not identify combined common and rare variant associations (SKATjoint) that were absent in testing the rare or common variants alone and therefore show this data in the supplemental material only (Additional file 1: Table S4, S5, S6).

#### *NCF4* rare variant carriers present with lower cytokine production in response to PHA stimulation

**Fig. 4:**
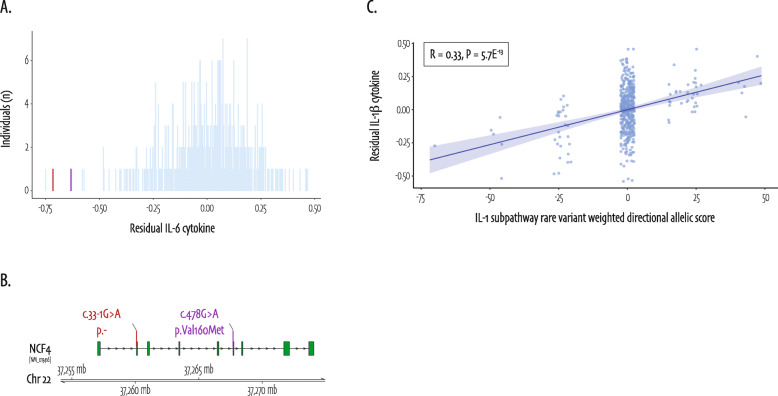
*NCF4* and IL-1 subpathway rare variants and effects on cytokine production. **a** *NCF4* rare variant carriers are in the lowest extreme of PHA-induced residual (corrected for age and sex) IL-6 cytokine production of the histogram distribution. Individuals without *NCF4* rare variants are shown in skyblue, individuals carrying a rare variant contributing to the association in red and purple. In **b**, the variants contributing to the association are annotated in the same red and purple colors on the most abundant transcript of *NCF4* (NM_013416). **c** A combined weighted directional allelic score for IL-1 subpathway rare variants in correlation with LPS-induced residual IL-1β cytokine production that is accompanied by a Spearman R of 0.33, *P* value = 5.7E^−13^

Our gene-level analysis significantly associated rare genetic variants in *NCF4* with cytokine production of both IL-1β and IL-6 in response to PHA stimulation (SKATO _adj_*P* value = 0.02 and 2.88E^−05^ respectively, Fig. 2). The association with IL-6 cytokine was based on two variants in two individuals: (1) a splice acceptor variant c.33-1G>A that has never been observed before, and splice predictions indicate that the probability that this canonical position is used as a splice acceptor site is decreased by 98.1%; and (2) a previously described known missense variant [43], c.478G>A, located in a region that is intolerant to variation [44]. The SKAT rho value of 1, indicated a unidirectional association, which is reflected by the fact that the individuals carrying these two variants present with extremely low PHA-induced IL-6 cytokine production (Fig. 4).

We identified another rare variant association between LPS-induced IL-6 cytokine production and *CASP1* (SKATO _adj_*P* value = 3.18E^−05^, Fig. 2b), based on five variants in 15 individuals. Our allelic score follow-up was unable to detect a significant correlation between age- and sex-corrected (residual) LPS-induced IL-6 cytokine production (Spearman R = 0.08, *P* value = 0.09), suggesting that one outlier individual, was driving the entire association (Additional file 2: Fig. S3A).

Common variants on the gene level were exclusively associated with the *C. albicans* in vitro stimulus. Specifically, common variants in *IL36A* and *IL38* were significantly associated with the production of both IL-1β and IL-6 (*IL36A* SKAToC _adj_*P* value = 0.04 and 3.69E^−03^; *IL38* SKAToC _adj_*P* value = 9.16E^−03^ and 8.21E^−03^, Fig. 2).

#### Rare variants in IL-1 subpathway genes combined are bidirectionally associated to LPS-induced IL-1β cytokine

On the subpathway level, *i.e.*, multiple genes that represent an immunological cascade in the IL-1-mediated inflammatory response, we identified a significant bidirectional burden of rare genetic variants in IL-1 subpathway genes combined with LPS-induced IL-1β cytokine production (SKATO _adj_*P* value = 7.12E^−03^, Fig. 2a). We translated this set-based association into an allelic score, by multiplying the IL-1 subpathway underlying rare variant dosages with the same allele frequency-based directional weights as used in the SKAT. Figure 4c shows a strong correlation between residual LPS-induced IL-1β cytokine production and the IL-1 subpathway allelic score (Spearman R = 0.33, *P* value = 5.7E^−13^). Besides this, LPS-induced IL-6 cytokine production was significantly associated with rare variants in the inflammasome subpathway (SKATO _adj_*P* value = 3.83E^−03^, Fig. 2b), reflected by a significant but modest correlation between the inflammasome allelic score and residual LPS-induced IL-6 cytokine (Spearman R = 0.21, *P* value = 7.2E^−06^, Additional file 2: Fig. S3B).

Finally, common variants in IL-30s subpathway genes were significantly associated with the production of both IL-1β and IL-6 cytokine in response to *C. albicans* stimulation (SKAToC _adj_*P* value = 1.66E^−03^ and 1.81E^−04^ respectively, Fig. 2). In our allelic score follow-up, the correlation between residual cytokine production and IL-30s common variants was stronger and more significant in IL-6 as compared to IL-1β cytokine (IL-1β Spearman R = 0.15, *P* value = 0.001; IL-6 Spearman R = 0.21, *P* value = 6.5E^−06^, Additional file 2: Fig. S3C, S3D). The difference in correlation reflects the SKAT association strengths.

#### Anti-inflammatory rare variant carriers show increased *S. aureus*-induced IL-6 cytokine production

Collapsing variants into anti- and pro-inflammatory groups on the inflammatory level, we detected two strong rare variant associations with IL-6 cytokine production upon stimulation. Rare variants in genes with pro-inflammatory effects were bidirectionally associated with LPS-induced IL-6 cytokine production (SKATO _adj_*P* value = 1.99E^−03^, Fig. 3b). The high degree of bidirectionality in this association (*i.e.*, variants leading to either lower or elevated cytokine levels, as indicated by SKAT rho value = 0) is reflected by the significant correlation between residual IL-6 cytokine in response to LPS stimulation and the pro-inflammatory allelic score (Spearman R = 0,36, *P* value = 2.1E^−15^, Additional file 2: Fig. S3E). On the other hand, rare variants in anti-inflammatory genes were unidirectionally associated with *S. aureus*-induced IL-6 cytokine production (SKATO _adj_*P* value = 6.71E^−03^, Fig. 3b). Figure 3c zooms in on this association, highlighting that individuals carrying a rare variant in an anti-inflammatory gene present with a higher residual *S. aureus*-induced IL-6 cytokine production as compared to non-carriers (Wilcoxon rank sum *P* value = 0.003).

Common variant associations were again exclusively observed with *C. albicans*-induced cytokine production (Fig. 3). Namely, common variants in anti-inflammatory genes were associated with IL-1β and even stronger with IL-6 cytokine (SKAToC _adj_*P* value = 1.87E^−03^ and 5.75E^−04^ respectively), and pro-inflammatory common variants exclusively with IL-6 cytokine production in response to *C. albicans* stimulation (SKAToC _adj_*P* value = 0.02). For all three associations, we confirmed the significant correlation between *C. albicans*-induced residual cytokine and set-based allelic scores (Additional file 2: Fig. S3F, S3G, S3H).

#### Common variants are exclusively associated with the immunological response to *C. albicans*

**Fig. 5:**
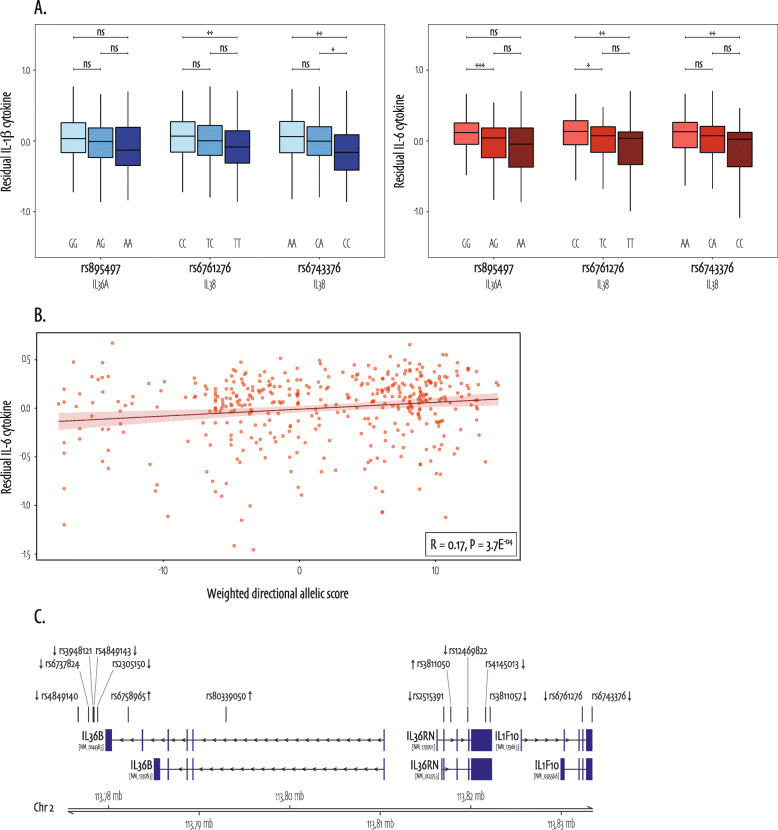
Coding and non-coding common variant set associations with *C. albicans*-induced cytokine production. **a** Residual (corrected for age and sex) IL-1β (left in blue) and IL-6 (right in red) cytokine production for coding SNPs in *IL36A* and *IL38* decreases over the genotype categories. For all plots, the ancestral allele is the minor allele and thus the genotype categories are ordered from left to right: homozygous alternative (IL-1β in light-blue and IL-6 in light-red), heterozygous (IL-1β in mid-blue and IL-6 in mid-red), homozygous ancestral (IL-1β in light-blue and IL-6 in light-red). Significant Wilcoxon rank sum *P* values are observed for IL-1β rs6761276 CC vs TT = 0.008, IL-1β rs6743376 AA vs CC = 0.001, IL-1β rs6743376 CA vs CC = 0.04, IL-6 rs895497 GG vs AG = 8.2E-04, IL-6 rs6761276 CC vs TC = 0.01, IL-6 rs6761276 CC vs TT = 0.005, and IL-6 rs6743376 AA vs CC = 0.003. Annotation: * = Wilcoxon rank sum *P* value < 0.05; ** = Wilcoxon rank sum *P* value < 0.01; *** = Wilcoxon rank sum *P* value < 0.001. **b** Visualizes the significant Bonferroni-adjusted association between coding and non-coding common variants in *IL38* variants and *C. albicans*-induced IL-6 cytokine production by means of a weighted, directional, allelic score summarizing the combined effect of all variants in the set in correlation with IL-6 cytokine. The straight line represents the linear model equation using method “lm”’ with standard error of 0.95, and the R of 0.17 represents the Spearman correlation coefficient with accompanying *P* value = 3.7E^−04^. **c** All common and non-coding SNPs with significant linear model *P* values (15 out of 41) are shown on top of transcripts that fall in the region of our *IL38* (gene name *IL1F10*) set

Over all levels of grouping, we observed associations between common variants and *C. albicans*-induced cytokine production, reflecting a common variant signature in this immunological response. For the underlying variants in the gene-level associations (*IL36A*: rs895497; *IL38*: rs6761276 and rs6743376), we observed that the alternative allele presented with (1) a higher frequency and (2) a higher *C. albicans*-induced residual cytokine production as compared to the ancestral (reference) allele, suggesting positive selection of the alternative or variant allele over the ancestral allele. Figure 5a shows that for each of these variants, cytokine production (IL-1β in blue and IL-6 in red) clearly decreases in the heterozygous and even more in the homozygous variant carriers. Specifically, significant differences were observed between homozygous reference and homozygous alternative genotypes for *IL38* variants rs6761276 and rs6743376 and both cytokines (Wilcoxon rank sum *P* values: IL-1β rs6761276 = 0.008, rs6743376 = 0.001; IL-6 rs6761276 = 0.005, rs6743376 = 0.003). In addition, we observed a significant difference between rs6743376 heterozygous carriers and homozygous reference only in IL-1β levels (Wilcoxon rank sum *P* value = 0.04), whereas rs6761276 heterozygous carriers and homozygous alternative presented with significantly different IL-6 levels (Wilcoxon rank sum *P* value = 0.01). And finally, for rs895497 (*IL36A*), heterozygous carriers presented with significantly higher IL-6 cytokine as compared to homozygous reference (Wilcoxon rank sum *P* value = 8.2E^−04^).

Accumulating evidence highlights a role for common non-coding genetic variation in human health [38, 39], in inflammatory responses [45, 46], and even specifically in innate immune responses [47–49]. While the rest of our study focused on coding variants, *i.e.*, variants that likely have a direct effect on protein function, we therefore additionally aimed to gain insight into the impact of non-coding common variants. Consequently, we expanded our significant coding common variant associations with previously published genotyping data from the same (500FG) cohort containing coding and non-coding common genome-wide genetic variation [18]. Coding and non-coding common variants (cohort AF ≥ 5%) in *IL36A*, *IL38*, IL30s subpathway, anti-inflammatory, and pro-inflammatory sets were pruned for LD, after which they subjected to the same SKAT. We identified a significant Bonferroni-adjusted association for these *IL38* variants with *C. albicans*-induced IL-6 cytokine production (SKAToC _adj_*P* value = 0.04). Figure 5b visualizes this association by means of a positive significant Spearman correlation of 0.17 (*P* value = 3.7E^−04^) between the *IL38* allelic score and residual cytokine production. Figure 5c shows all significant SNPs falling in the *IL38* genic region and 50 kb upstream sequence which includes other IL-1 pathway genes *IL36B* and *IL36RN*. To explore the individual contribution of non-coding common variants in significant *IL38* set, we organized linear model single SNP effect estimates by direction and significance (Additional file 1: Table S11). rs80339050, located in an intron of *IL36B* ~ 38kB upstream of *IL38* (*IL1F10* gene), presented with the largest significant effect estimate on *C. albicans*-induced IL-6 cytokine production. This SNP falls into multiple transcription factor binding sites and may therefore exert a regulatory function. In addition, public eQTL databases identified rs80339050, just as rs6761276 and rs6743376 our coding associated SNPs in *IL38*, as an eQTL for IL-1 pathway genes in *Listeria monocytogenes*, *Salmonella typhymurium*, and non*-*infected macrophages [42].

## Discussion

In this study, we identified and characterized rare and common genetic variants in genes related to the IL-1 pathway and determined their impact on the inter-individual variability of stimulus-induced in vitro cytokine responses in whole blood from healthy individuals. By employing grouping strategies over various levels of magnitude, from gene to subpathway to inflammatory level, we assessed the contribution of rare and common variants, and thereby highlighted stimulus- and frequency-specific variant set involvement in IL-1β and IL-6 cytokine responses. An intrinsic issue with rare variants is their low frequency, resulting in limited power for association testing, in particular for healthy continuous phenotypes [50]. This power issue can be addressed, by using prior knowledge on the biological effects of the genes studied to combine variants into functional sets, thereby increasing the number of variants per test and reducing the number of tests that need to be performed.

We identified two rare variant associations with three cytokine-stimulus combinations in the gene-level variant sets; *CASP1* with LPS-induced IL-6 cytokine production, and *NCF4* with PHA-induced IL-1β and IL-6 production. The fact that we observed a burden of *CASP1* rare variants with IL-6 production and not with IL-1β is surprising, as CASP-1 protein is most known for cleavage of the inactive mediators IL-1β, IL-18, and IL-33 into their active form [2]. However, abnormal pyroptosome formation and impaired nuclear localization independent of the enzymatic activity of CASP-1 in processing pro-IL1β into active IL1β was previously observed [51]. Importantly, in our allelic score follow-up, we were unable to detect a significant correlation, suggesting that this association was mainly driven by a single individual with extremely low IL-6 cytokine production (Additional file 2: Fig. S3A). Repeating the SKAT without this individual subsequently abolished the significance, indicating that this result may suggest a false positive association driven by this outlier or a complex gene-gene or gene-environment interaction leading to the unlikely combination of extremely low cytokine production and homozygous genotype of rs61751523. This example therefore motivates the careful follow-up of significant (rare) variant associations. In contrast, we identified a robust unidirectional burden of rare variants in *NCF4* and PHA-induced IL-6 cytokine production. The *NCF4* gene encodes the NCF4 protein which is part of the cytoplasmic unit of the NADPH (nicotinamide adenine dinucleotide phosphate) oxidase enzyme system involved in phagocytosis [52]. It is well known that mutations in the NADPH complex can lead to dysregulated cytokine production in the primary immunodeficiency chronic granulomatous disease (CGD) [53, 54]. The consequences of these mutations can be cell type specific and are known to cause phenotypes deviating from classic CGD [55], which suggests a high degree of complexity in the interaction between NADPH and cytokine production. Adding to this complexity, our study revealed an association between *NCF4* rare variants and lower IL-6 cytokine in response to PHA, and therefore mechanistically requires further investigation.

In addition to the above discussed gene-level findings, this study highlights the advantage of using larger functionally defined groups. Specifically, rare variants in the IL-1 subpathway were bidirectionally associated with LPS-induced IL-1β production, and we identified a unidirectional burden of rare variants in anti-inflammatory genes combined with *S. aureus*-induced IL-6 cytokine production, even though the individual genes part of these two sets did not produce an association with the respective phenotype. The burden of anti-inflammatory rare variants is interesting, as more than half of the anti-inflammatory genes are autophagy genes, supporting the notion that defective autophagy results in increased cytokine production, with increased inflammatory disease severity, such as CGD and inflammatory bowel disease—especially colitis observed in Crohn’s disease—as a consequence [53, 56]. Additional diseases characterized by dysregulated inflammation in which defects of autophagy, and subsequently higher cytokine production, are also SLE and sarcoidosis [57, 58]. Interestingly, *S. aureus*-mediated inflammatory effects have been also suggested to play a role in Wegener’s granulomatosis, and it would be tempting to speculate that this effect is stronger in individuals with certain mutation in genes of IL-1 pathway [59].

In contrast to rare variants, the *C. albicans* stimulus-specific common variant associations identified in this study constituted variant sets over multiple grouping levels. The importance of common genetic variants in the innate immune pathway in immunological defense against *C. albicans* is supported by existing literature [60, 61]. However, in the previously published study using the same cohort, none of the IL-1 pathway genes were significantly associated with *C. albicans*-induced cytokine production [9]. The GWAS summary statistics show nominal significance for these particular variants, but do not reach genome-wide significance, highlighting the advantage of our targeted approach and set-based framework here (Additional file 1: Table S12). An additional independent validation by means of exact replication of cytokine QTL would be most favorable, but remains challenging as they can be cell type and context specific as previously shown [10, 62]. Nonetheless, the stimulus-specific variant frequency effect is noteworthy shown, especially in combination with the phenomenon that the ancestral allele in individual variants presents with lower IL-6 cytokine upon *C. albicans* stimulation (Fig. 5a). This observation may be interesting in light of the co-evolution of commensal yeast species and humans as oral candida infections appear to have been described as early as the second century [63]. Next to highlighting the role for coding common variants, we expanded our study using non-coding common variants in the same cohort. The significant association of coding and non-coding common variants in *IL38* supports the importance of considering a combined effect of multiple common variants. How these common non-coding variants may possibly impact gene expression levels requires dedicated follow-up studies and remains speculative so far. For instance, rs80339050, the SNP in our *IL38* set with highest effect estimate, falls into multiple transcription factor binding sites and may therefore exert a regulatory function. Long-range contact assessment can help to understand local genome architecture, although this could be cell type and context specific, substantiating the urge for studying the impact of non-coding variants in immunity [64]. Interestingly, the same variant was previously shown to act as an eQTL of IL-1 pathway genes after in vitro bacterial stimulations with *Listeria monocytogenes*, *Salmonella typhymurium*; suggesting that this locus may have bona fide regulatory effects in the broad spectrum of human pathogen responses. Indeed, IL-38 is an important regulatory cytokine for inflammatory response in general and IL-6 pathway in particular [65], and our data suggest such effects on the inflammation induced by human fungal and bacterial pathogens alike.

Our study cohort is one of the largest to date in which extensive immunophenotyping experiments have been performed [8, 9, 11]. The associations described here are based on cytokine production by whole blood, *i.e.*, a mix of immune cell subtypes, warranting the cross-regulation between different cell types in determination of the final immune response. The investigated stimuli were chosen as representatives for an array of microbial infections. Future efforts investigating a broader array of pathogens, as well as the specific contribution of immune cell subtypes, would be highly interesting. Nevertheless, potential limitations of this study include the relatively small sample size for genetic studies and cohort characteristics (restricted age distribution and residency), and replication in a larger cohort for validation is favorable. In order to substantiate our findings, we applied a threefold validation approach, in which all non-synonymous significant associations were validated by permutation tests, by extreme outlier analysis, and by comparing the burden of synonymous rare variants. Our synonymous validation did however produce two borderline significant results in other sets, which could either uncover possible false positive associations, potentially due to the limitations of this study cohort, or could be true findings as not all synonymous variation is fully neutral [66]. Secondly, despite the cost-effectiveness of MIP sequencing (*e.g.*, ±€25, per sample for the IL-1 panel), larger intronic or non-coding regions are not sequenced and as such escape analysis. The potential of using whole genome sequencing data to investigate the role of all rare coding and non-coding genetic variation, thereupon, seems promising, but the larger targets may require an even bigger sample size. Thirdly, the SKAT is powerful, but computes only set-wise association *P* values and does not provide single-variant effect estimates, neither does it provide direction in terms of positive/negative effects or increased/decreased risk. Our customized allelic score illustrates the set-based effect, but is most likely weaker as compared to the SKAT output, as we cannot exclude potential heterogeneity or interaction of variants in a set. Furthermore, the contribution of single variants to a phenotype is difficult to estimate and as such the clinical applicability remains complex and requires more in-depth functional follow-up. Lastly, we present a framework that allows one to analyze the burden of rare and common variants and their effect on inter-individual immune-response differences, which provides initial insights for fundamental biology or disease understanding. The fact that we use cytokine responses in a cohort of healthy individuals could be a possible explanation for the absence of even stronger effects, and similar studies are warranted in disease cohorts. This, we have recently demonstrated by the identification of six individuals carrying four different rare variants in *IL37* that present with a more severe clinical form of gout [67].

## Conclusions

In conclusion, this study shows that common and rare genetic variation in genes of the IL-1 pathway in functionally defined groups over various levels, differentially influence in vitro IL-1β and IL-6 cytokine responses induced by various stimuli. In particular, our rare variant associations in *NCF4* with specific stimulation-induced cytokine responses are in line with previously published defects known to contribute to the phenotypic presentation of inflammation-mediated diseases such as CGD. Furthermore, a bidirectional burden of rare variants in IL-1 subpathway genes combined is correlated with IL-1β cytokine production levels. And finally, rare variants in genes encoding proteins with known anti-inflammatory function result in increased cytokine production, which is in line with proposed autophagy defects resulting in aggravated inflammatory disease severity. In contrast, the identified common variant associations for *C. albicans*-induced cytokine responses, replicates other common variant effects for this pathogen.

Altogether, this study provides insights into genetic variant effects on IL-1 mediated immunological cascades, potentially affecting the clinical presentation of a particular inflammatory disease. On a broader perspective, these findings could be used to prioritize genes or variants in inflammation-mediated diseases that share clinical similarities, but for which no single genetic defect has been identified to date. The framework presented here can be applied to other (molecular) phenotypes of interest and therefore has the potential to contribute to better understanding of unresolved, complex traits and diseases.

## Supplementary Information

**Additional file 1: Supplementary Tables. Table S1.** Variant grouping strategies of 48 Interleukin-1 pathway related genes. **Table S2.** Molecular Inversion Probes (MIPs) covering all coding exons of 48 genes of the Interleukin-1 pathway. **Table S3.** Non-synonymous variant list. **Table S4.** Gene-level SKAT output. **Table S5.** Subpathway-level SKAT output. **Table S6.** Inflammatory-phenotype level SKAT output. **Table S7.** Validation SKAT output – permutations. **Table S8.** Synonymous variant list. **Table S9.** Validation SKAT output – synonymous. **Table S10.** Validation SKAT output – extreme outliers. **Table S11.** Common coding and non-coding IL38 set single SNP linear model parameters. **Table S12.** GWAS P-value versus SKAT _adj_*P*-value.

**Additional file 2: Supplementary Figures. Figure S1.** Baseline characteristics of healthy individuals (*n* = 463) included in analysis. **Figure S2.** Average coverage depth per gene and overall for healthy individuals (*n* = 463) included in analysis. **Figure S3.** Correlation plots of significantly associated sets.

## Data Availability

All demographic, immunophenotyping, and genotyping data from the 500FG cohort used in this study is publicly available on the BBMRI-NL archive (https://hfgp.bbmri.nl/) [18], and Human Functional Genomics Project website (http://www.humanfunctionalgenomics.org/site/) [17]. In compliance with the original consent forms, we cannot share identifiable information; therefore, the raw sequencing data cannot be made publicly available. The following data are included in this published article and its supplementary information files: all variants called in MIP sequencing data based on the IL-1 panel (Additional file 1: Table S3, S8 for non-synonymous and synonymous variants respectively), all association results (Additional file 1: Table S4, S5, S6 for non-synonymous SKAT test output, and Additional file 1: Table S9 for synonymous SKAT test output). Code for processing and filtering MIP-based sequencing data are extensively explained in the “Methods” section of this manuscript and available in a public GitHub repository (https://github.com/RosanneVanDeuren/mip-RsCh-pipe) [68]. The source code from the R packages used in this study are freely available online via CRAN (https://cran.r-project.org/) [33] or Bioconductor (https://bioconductor.org) [34].

## Abbreviations

_adj_*P* value: Bonferroni-adjusted *P* value
AF: Allele frequency
AoSD: Adult-onset Still’s disease
BAQ: Base Alignment Quality
BWA-MEM: Burrows-Wheeler Aligner
*C. albicans*: *Candida albicans*
CAPS: Cryopyrin associated periodic syndromes
CFU: Colony-forming unit
CGD: Chronic granulomatous disease
cQTL: Cytokine quantitative trait loci
dbSnp: Single nucleotide polymorphism database
DIRA: Deficiency of IL-1 receptor antagonist
DNA: Deoxyribonucleic acid
eQTL: Expression quantitative trait loci
ELISA: Enzyme-linked immunosorbent assay
ExAc: Exome Aggregation Consortium
FG: Functional Genomics
GATK: Genome Analysis Toolkit
gnomAD: Genome Aggregation Database
GWAS: Genome-Wide Association Studies
HFGP: Human Functional Genomics Project
IL-1β: Interleukin-1β
IL-6: Interleukin-6
IL: Interleukin
LD: Linkage disequilibrium
LPS: Lipopolysaccharide
MAF: Minor allele frequency
MIP: Molecular Inversion Probe
NADPH: Nicotinamide adenine dinucleotide phosphate
NCBI RefSeq: National Center for Biotechnology Information Reference Sequence
PHA: Phytohaemagglutinin
PMNs: Polymorphonuclear cells
QTL: Quantitative trait loci
QUAL: Quality parameter in vcf
R^2^: Correlation metric
RA: Rheumatoid arthritis
Radboudumc: Radboud university medical center
RNA: Ribonucleic acid
ROS: Reactive oxygen species
RVBA: Rare variant burden analysis
*S. aureus*: *Staphylococcus aureus*
SKAT: Sequence Kernel Association Test
SKATjoint: SKAT common and rare variants
SKATO/SKAT-O: Linear combination of the Burden Test and SKAT with optimal weights
SKAToC: SKAT only common variants
SLE: Systemic lupus erythematodes
SNP: Single nucleotide polymorphism
vcf: Variant Call Format
