# Supplementary material for: Impact of rare and common genetic variation in the interleukin-1 pathway on human cytokine responses
Source: Genome Med. 2021 May 25;13:94. doi: 10.1186/s13073-021-00907-w (PMC8145796; doi:10.1186/s13073-021-00907-w)
Supplement: Supplementary file 2 — Additional file 2: Supplementary Figures. Figure S1. Baseline characteristics of healthy individuals (n = 463) included in analysis. Figure S2. Average coverage depth per gene and overall for healthy individuals (n = 463) included in analysis. Figure S3. Correlation plots of significantly associated sets. [file 13073_2021_907_MOESM2_ESM.pdf]

A.

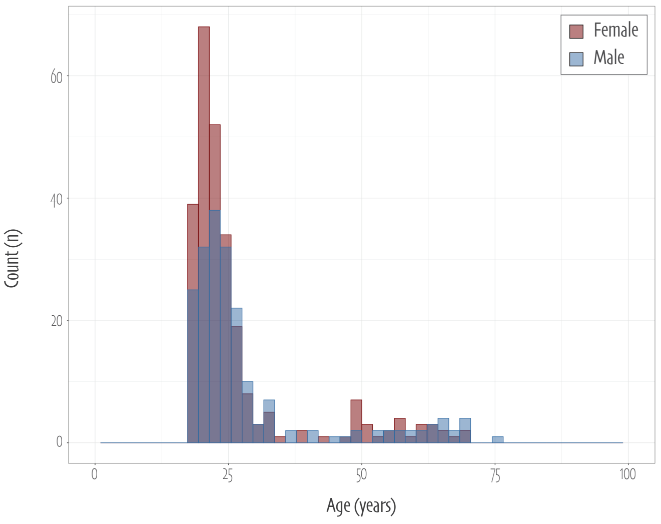

B.

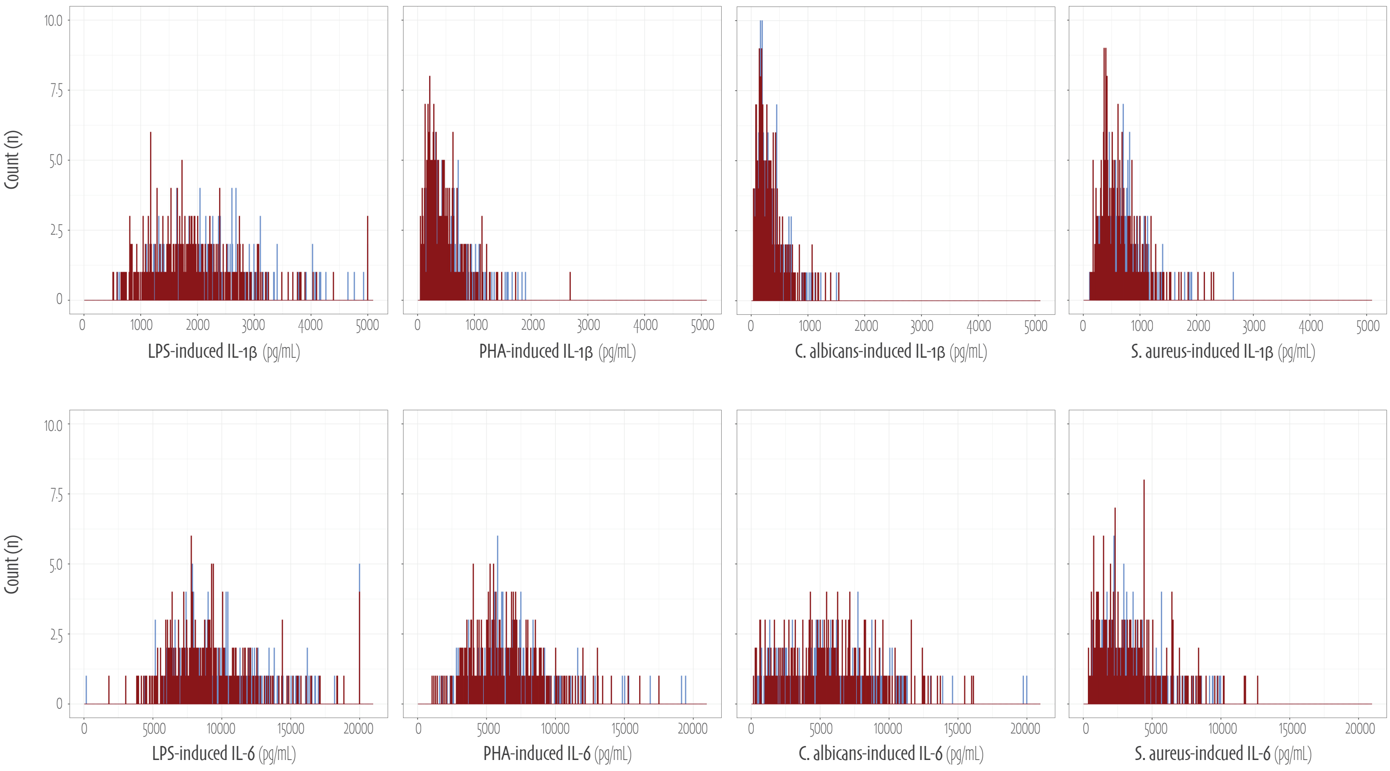

Fig. S1: Baseline characteristics of healthy individuals (n=463) included in analysis. (A) Shows the age distribution of all individuals included in analyses classified by gender (mean age - Female=26.66, Male=28.84; median age - Female=22, Male=24). **(B)** Shows the IL-1β and IL-6 in vitro cytokine production in response to LPS (100ng/mL), PHA (10μg/mL), C. albicans (10^6 CFU/mL), S. aureus ($1x10^6$/mL), classified by gender.

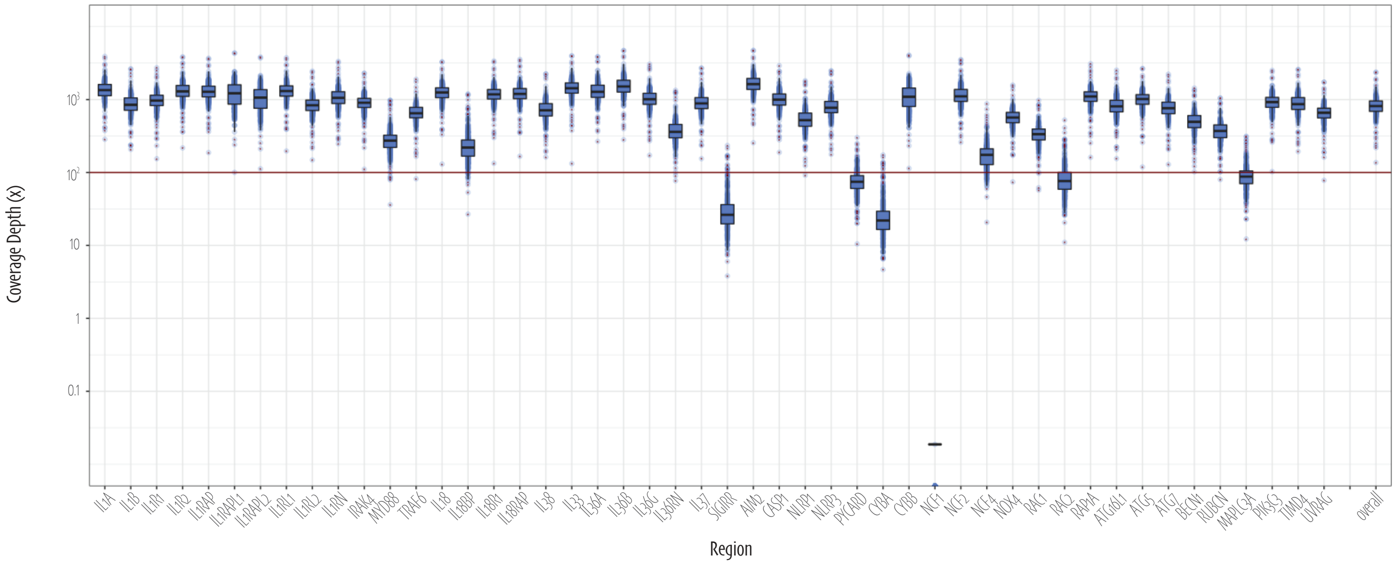

Fig. S2: **Average coverage depth for healthy individuals (n=463) included in analysis.** The average coverage for each individual per gene (and overall on the most right) is visualized in separate jitter plots, overlayed by boxplots, where boxes contain a median line, edges at 25th and 75th percentile, and default whiskers (1.5 * InterQuartileRange). Logarithmic y-axis, with a horizontal red line representing the analysis inclusion coverage cut-off of 100x.

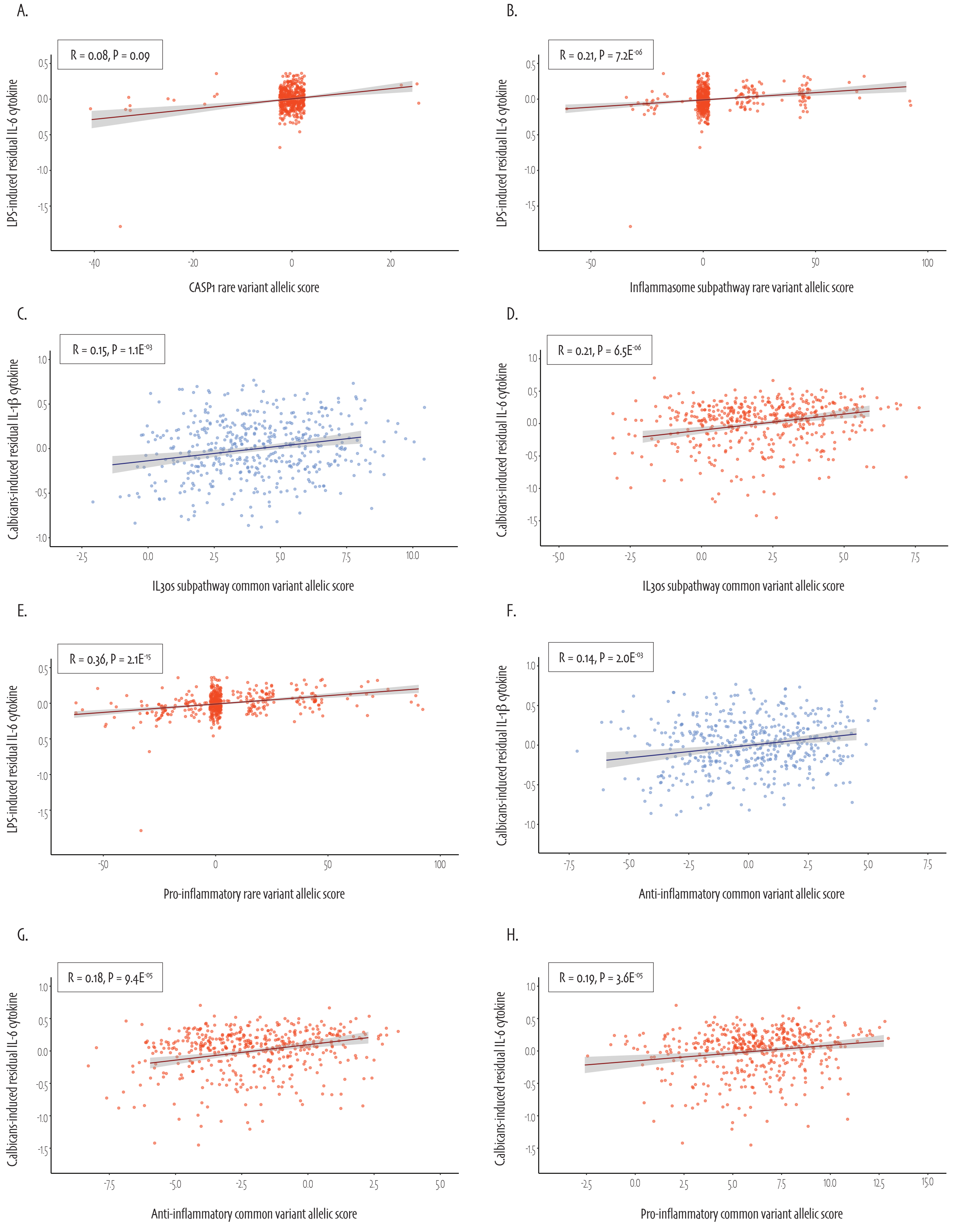

Fig. S3: Correlation plots of significantly associated sets. Stimulation-induced residual (corrected for age and sex) cytokine production in correlation with set-based, directional, allele frequency weighted allelic scores for significant bi-directional rare variant and common variant (variant n > 2) associations. **(A)** LPS-induced IL-6 residual cytokine production in correlation with CASP1 rare variant (n=5) allelic score. **(B)** LPS-induced IL-6 residual cytokine production in correlation with inflammasome subpathway rare variant (n=42) allelic score. **(C)** C. albicans-induced IL-1β residual cytokine production in correlation with IL30s subpathway common variant (n=7) allelic score. **(D)** C. albicans-induced IL-6 residual cytokine in correlation with IL30s subpathway rare variant (n=7) allelic score. **(E)** LPS-induced IL-6 residual cytokine production in correlation with pro-inflammatory rare variant (n=102) allelic score. **(F)** C. albicans-induced IL-1β residual cytokine production in correlation with anti-inflammatory common variant (n=10) allelic score. **(G)** C. albicans-induced IL-6 cytokine production in correlation with anti-inflammatory common variant (n=10) allelic score. **(H)** C. albicans-induced IL-6 residual cytokine production in correlation with pro-inflammatory common variant (n=16) allelic score.
